# Design and Experimental Analysis of a Multi‐Scale Fresh Tea Leaf Rotary Drum Screen Based on the DEM‐RSM Method

**DOI:** 10.1002/fsn3.71983

**Published:** 2026-06-04

**Authors:** Xu Zhang, Rongyang Wang, Zhongyou Zhou

**Affiliations:** ^1^ Huzhou Vocational and Technical College College of Intelligent Manufacturing Huzhou China

**Keywords:** discrete element simulation, fresh tea leaves, response surface methodology, rotary drum screen, separation efficiency

## Abstract

Conventional rotary drum screens for fresh tea leaf classification suffer from persistent limitations, including screen aperture clogging, particle entanglement, and suboptimal operating parameter tuning, which impede the efficient and precise sorting of multi‐grade fresh tea leaves. To address these challenges, this study presents a high‐performance rotary drum screen design optimized via a coupled Discrete Element Method (DEM) and Response Surface Methodology (RSM) framework, validated through systematic experimentation. DEM simulations were employed to evaluate two screen aperture configurations: the conventional sequential “small‐medium‐large” layout and a novel alternating “small‐medium‐small‐medium‐large” design. Results demonstrated that the proposed layout effectively mitigates particle entanglement and reduces clogging, yielding superior screening performance. Single‐factor experiments were conducted to define feasible operating ranges for drum rotational speed, inclination angle, and feed rate. Subsequent RSM experiments quantified the interactive effects of these parameters and established a regression model for separation efficiency. Optimization via Design‐Expert software identified the optimal operating conditions: a rotational speed of 13 r/min, an inclination angle of 5.5°, and a feed rate of 300 fresh tea leaves (0.132 kg). Under these conditions, the average separation efficiency reached 70.49%. The DEM‐RSM coupled approach presented herein provides a robust theoretical foundation for the structural innovation and parameter optimization of fresh tea leaf classification equipment, advancing the standardization and efficiency of primary tea processing.

## Graphical abstract

Through discrete element simulation and test verification, a new type of sieve arrangement drum sorting equipment is determined, single factor test, response surface method test and test optimization, and the optimal parameter sorting rate of 70.49% is obtained, which provides a basis for equipment research and development and provides a prospect for subsequent research.

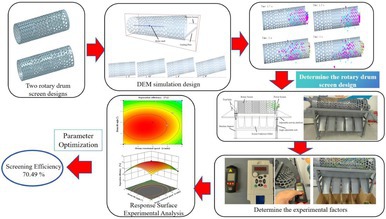

## 1 Introduction

As Tea is a cornerstone economic crop and cultural symbol in China, with its final quality inextricably linked to every stage of processing. Among these, the classification of fresh tea leaves represents a critical unit operation in primary tea manufacturing (Ren et al. 2025; Gan et al. 2024). Multi‐grade fresh tea leaves—categorized as single leaves, one‐bud‐one‐leaf, one‐bud‐two‐leaf, and one‐bud‐multi‐leaf—exhibit distinct size and maturity characteristics that dictate subsequent processing routes, as well as the grade and economic value of the final product (Zhang et al. 2025). High‐quality tender single leaves and one‐bud‐one‐leaf materials are utilized for premium green and black teas, while coarser one‐bud‐two‐leaf and multi‐leaf materials are primarily allocated to tea beverages and extracts. Therefore, the development of efficient and accurate multi‐grade fresh tea leaf classification technologies is pivotal to enhancing processing standardization and elevating the industrial added value of the tea sector (Hu et al. 2025; Liu et al. 2024).

Rotary drum screens are the dominant technology for fresh tea leaf classification in current industrial practice (Dai et al. 2023; Xue et al. 2020). Their performance is contingent upon screen aperture size and arrangement; however, existing equipment faces significant technical bottlenecks. First, the widely adopted conventional “small‐medium‐large” aperture layout is prone to particle entanglement and interlocking, leading to severe screen clogging and reduced screening efficiency (Li et al. 2024; Wang et al. 2024; Gan et al. 2023). Second, key operating parameters—including drum rotational speed, inclination angle, and feed rate—are typically determined empirically, lacking systematic multi‐factor optimization. This results in insufficient classification accuracy and poor operational stability, failing to meet the refined sorting requirements of multi‐grade fresh tea leaves (Wang 2025; Song et al. 2020). Furthermore, fresh tea leaves are flexible sheet materials with highly sensitive physical properties, rendering them susceptible to damage and deformation during processing. Traditional experimental methods are unable to accurately capture their dynamic motion behavior during screening (Huang 2025; Kuang et al. 2026), further hindering equipment optimization and upgrading.

The Discrete Element Method (DEM) is a powerful numerical technique for simulating granular material motion, capable of replicating the dynamic characteristics of flexible particles during screening—including collision, tumbling, and aperture penetration—thereby providing visual and quantitative support for screen aperture design (Liu et al. 2025; Zhang et al. 2023, 2024). Response Surface Methodology (RSM) is a robust statistical approach for multi‐factor optimization, enabling the establishment of nonlinear relationships between input parameters and response values with a limited number of experiments, thus facilitating efficient identification of optimal parameter combinations (Dong et al. 2026; Chen et al. 2024, 2023). The coupled application of DEM and RSM offers a promising pathway to overcome the limitations of conventional design and optimization methods, supporting the precision development of fresh tea leaf rotary drum screens.

Against this backdrop, this study presents the design and experimental validation of a novel rotary drum screen tailored to multi‐grade fresh tea leaf classification, addressing core pain points of clogging, entanglement, and suboptimal parameter tuning in conventional equipment. First, dimensional parameters of single‐leaf, one‐bud‐one‐leaf, and one‐bud‐two‐leaf samples were measured to inform matched screen aperture specifications. Second, EDEM software was utilized to compare the screening performance of the conventional “small‐medium‐large” and the proposed “small‐medium‐small‐medium‐large” aperture layouts, verifying the superior performance of the novel design. Third, feasible operating ranges for drum rotational speed, inclination angle, and feed rate were determined via single‐factor experiments, and RSM was employed to establish a regression model for separation efficiency, enabling key parameter optimization. Finally, validation experiments confirmed the feasibility of the optimized scheme. This work aims to provide theoretical support and technical references for the structural innovation and parameter optimization of fresh tea leaf classification equipment, driving the advancement of primary tea processing technology toward high efficiency and refinement.

## 2 Materials and Methods

### 2.1 Sieve Hole Size Design

Fresh tea leaves are highly seasonal and prone to damage, withering, and deformation during physical experiments, posing challenges to repeated testing. Accordingly, this study employed 1:1 scale flexible fresh tea leaf models for primary screening experiments, with model design based on actual fresh tea leaf samples.

Multi‐grade fresh tea leaves for parameter calibration were manually harvested in April 2025 from a standardized tea plantation in Huzhou City, Zhejiang Province, China, including single‐leaf, one‐bud‐one‐leaf, and one‐bud‐two‐leaf samples. All samples were transported to the laboratory within 2 h post‐harvest to preserve their fresh state. Geometric dimensions were measured using a vernier caliper, and the mass of 20 parallel samples per grade was weighed to obtain average values, which served as the design basis for the 1:1 models. The models precisely replicated the geometric dimensions, mass, density, and contact mechanical properties of actual fresh tea leaves to ensure consistency between simulated and real screening conditions. Neither the calibration samples nor the 1:1 models underwent washing or drying procedures to avoid altering core physical properties and deviating from industrial classification processes.

**FIGURE 1:**
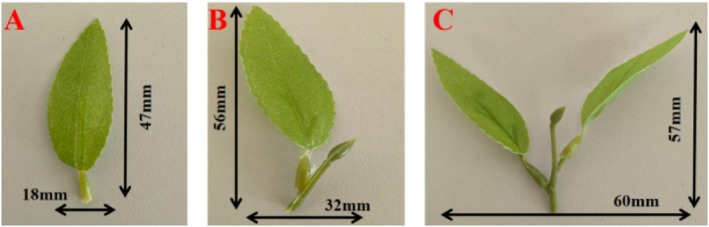
Multi‐scale tea green leaves.

Based on measured dimensions of experimental samples, drum screen apertures were designed in three specifications: small (20 × 50 mm), medium (35 × 60 mm), and large (60 × 60 mm). The drum had a diameter of 450 mm and a length of 1500 mm, as illustrated in Figure 1.

### 2.2 Working Principle of Drum Classification

Upon equipment startup, a gear motor drives the transmission gear via a coupling, rotating the drum screen body at a constant speed through a segmented chain. Support rollers rotate synchronously to provide stable support and reduce rotational resistance, ensuring uniform drum speed. The drum inclination angle (0°–10°) is adjusted via an angle adjustment mechanism based on tea leaf characteristics such as size, moisture content, and maturity: for high‐moisture, large‐sized leaves, a steeper angle is employed to accelerate material movement and prevent clogging; for tender, fragile leaves, a shallower angle is used to extend screening residence time and enhance classification accuracy.

Driven by the gravitational component of the drum's inclination angle, unscreened materials move slowly along the drum axis toward the oversize discharge port. During axial transport, materials are repeatedly lifted and dropped to ensure that small, qualified leaves mixed with coarse materials are fully screened, thereby improving separation efficiency. Ultimately, coarse old leaves and large impurities are discharged from the oversize port, achieving simultaneous classification and impurity removal of fresh tea leaves.

### 2.3 Discrete Element Simulation Analysis Method

#### 2.3.1 Drum Screen Design

**FIGURE 2:**
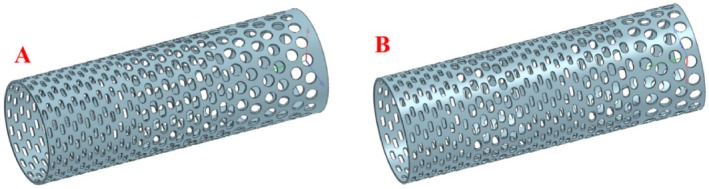
Layout design of drum screen holes.

Two screen aperture configurations were developed based on the principles of drum screening: the conventional “small‐medium‐large” layout (Figure 2A) and the novel “small‐medium ‐small‐medium‐large” layout (Figure 2B). The former is widely used in industrial practice and existing research, while the latter represents an innovative design proposed by the research team following preliminary theoretical analysis. EDEM discrete element analysis was utilized to simulate and evaluate the screening performance of both configurations. Apertures in both layouts are densely distributed in a regular array across the drum's outer surface, ensuring sufficient contact between materials and the screen surface during operation.

#### 2.3.2 Simulation Parameters

Discrete element simulations were performed using EDEM 2020 software. The Discrete Element Method was employed to simulate the screening process of multi‐grade fresh tea leaf particles entering the drum screen. The Hertz‐Mindlin (No Slip) contact model was selected for its high accuracy and computational efficiency in contact force calculations. The normal force component is based on Hertzian contact theory, while the tangential force model derives from Mindlin‐Deresiewicz contact theory, with both components incorporating damping terms related to the damping and restitution coefficients. Interactions between fresh tea leaves were modeled using a static elastic contact model, which effectively addresses curved surface contact between flexible particles. Interaction parameters between tea leaves and the drum screen wall were calibrated via preliminary tests and are presented in Table 1.

**TABLE 1:** Simulation parameter.

Item	Parameter	Value
Fresh Tea Leaves	Poisson's Ratio	0.4
	Shear Modulus	3.3e+6 Pa
	Solids Density	851.5 Kg/m^3^
Drum Screen Wall	Poisson's Ratio	0.3
	Shear Modulus	8e+7 Pa
	Solids Density	7850 Kg/m^3^
Fresh Tea Leaves—Fresh Tea Leaves	Coefficient of Restitution	0.12
	Coefficient of Static Friction	0.73
	Coefficient of Rolling Friction	0.03
Fresh Tea Leaves—Drum Screen Wall	Coefficient of Restitution	0.15
	Coefficient of Static Friction	0.22
	Coefficient of Rolling Friction	0.10

#### 2.3.3 Establishment of Simulation Model and Parameter Setting

Preliminary research by the team indicated that while 3D scanning of fresh tea leaf contours and particle filling can achieve 1:1 motion simulation (Zhang et al. 2023, 2024), this approach results in slow computation and low analysis efficiency. Given the primary objective of evaluating different aperture distributions, simplified ellipsoidal particles with dimensions matching screen aperture contours were used for analysis.

**FIGURE 3:**
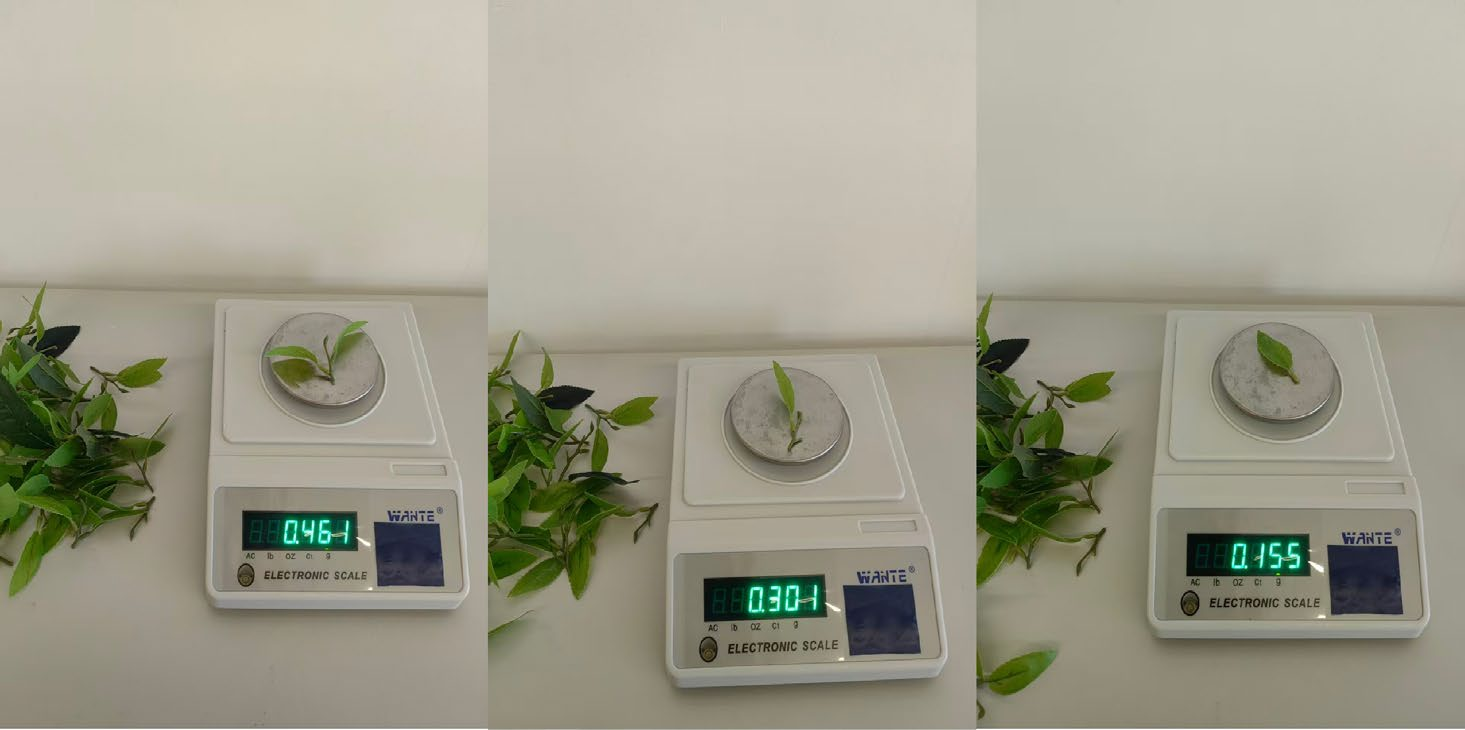
Weighing of the fresh tea leaf model.

Mass measurements of single‐leaf, one‐bud‐one‐leaf, and one‐bud‐two‐leaf models are presented in Figure 3. Twenty standard fresh tea leaf models per grade were weighed, yielding average masses of 0.158 (single‐leaf), 0.313 (one‐bud‐one‐leaf), and 0.468 g (one‐bud‐two‐leaf).

Multi‐grade fresh tea leaf particles were modeled as ellipsoids with major and minor axes strictly matching measured transverse and longitudinal dimensions: single‐leaf particles as 20 × 50 mm ellipsoids, one‐bud‐one‐leaf as 35 × 60 mm ellipsoids, and one‐bud‐two‐leaf as near‐spherical 60 × 60 mm ellipsoids. Particle density was back‐calibrated using 1:1 model mass data to ensure mass consistency with actual leaves, achieving dual matching of geometric scale and mass.

**FIGURE 4:**
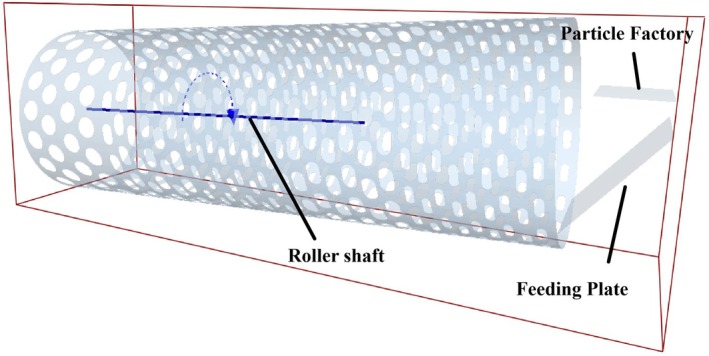
Simplified model of drum screen in DEM.

**FIGURE 5:**
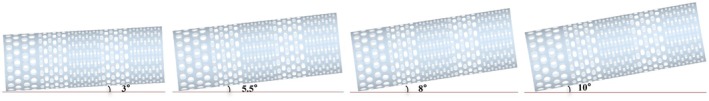
Different inclination angles of the drum.

To enhance computational efficiency, the drum screen model was simplified to consist of a drum and feed inlet, as shown in Figure 4. The drum rotational speed was set to 14 r/min. A virtual particle factory generated multi‐grade fresh tea leaf assemblies (200 particles per grade, generation rate 50 g/s, falling speed 0.3 m/s). The simulation time step ratio was set to 17%, with a total duration of 7 s. To avoid damage to tea leaves caused by internal guide plates in conventional drums, the design utilizes drum inclination adjustment instead. Tested inclination angles included 3°, 5.5°, 8°, and 10°, as illustrated in Figure 5.

#### 2.3.4 DEM Simulation Verification

A drum with the novel “small‐medium‐small‐medium‐large” aperture layout was selected for simulation and experimental validation under identical operating conditions: rotational speed 14 r/min, inclination angle 5.5°, feed rate 350 multi‐grade fresh tea leaves. Screening efficiency was used as the comparison metric.

DEM simulation predicted a screening efficiency of 61.23%, while physical experiments (three replicates) yielded an average efficiency of 63.80%. The relative error, calculated as follows:

(1) $$E=\frac{\left|{\bar{Y}}_{\mathrm{sim}}-{\bar{Y}}_{\exp}\right|}{{\bar{Y}}_{\exp}}\times 100\%$$

where E is the relative error; ${\bar{Y}}_{\mathrm{sim}}$ is the simulated screening efficiency; and ${\bar{Y}}_{\exp}$ is the experimental average efficiency, was found to be 4.03%. This value falls below the 5% allowable error threshold for DEM simulations in agricultural engineering, confirming the simplified model's high prediction accuracy and validity for subsequent analyses.

### 2.4 Statistical Analysis

Design‐Expert 13.0 was used for RSM design, regression modeling, ANOVA, and response surface visualization. All results are presented as mean ± standard deviation (SD). Mean values of replicates were used for core indicator calculations and regression model fitting, with SD characterizing data dispersion and experimental stability. Single‐factor experiments: Three independent replicates per factor level (*n* = 3). Box–Behnken design: 17 experimental groups, including 12 non‐central factor combinations and five central point replicates.

## 3 Results and Discussion

### 3.1 Screening Simulation Analysis

Blue particles represent single‐leaf, pink particles one‐bud‐one‐leaf, and yellow particles one‐bud‐two‐leaf. Two drum designs with identical geometry but different aperture distributions were analyzed under the same simulation timeline:

#### 3.1.1 Drum Inclination Angle of 3°

**FIGURE 6:**
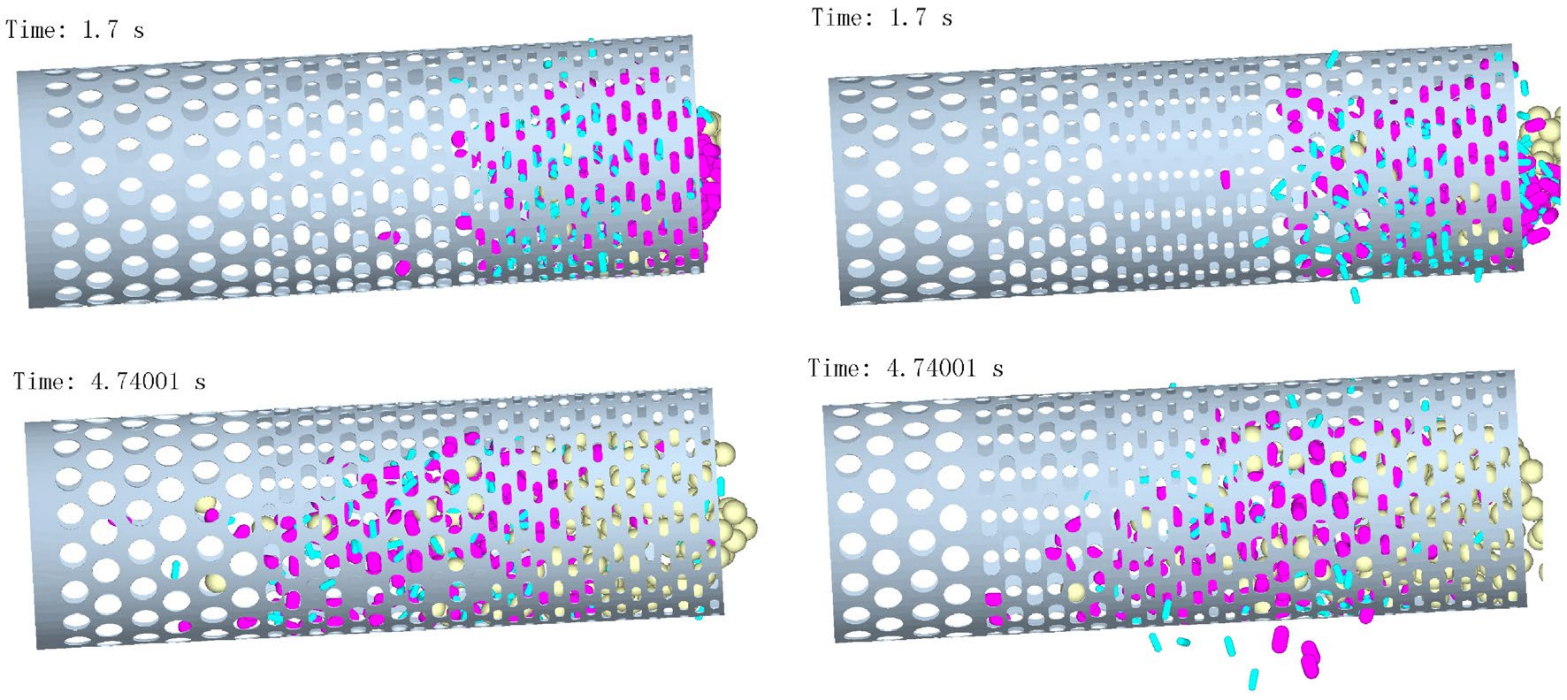
Particle motion when the inclination angle of the drum is 3°.

As shown in Figure 6, at *t* = 1.7 s, significant particle accumulation occurred in the small‐aperture section of the conventional (left) drum. Entanglement and interlocking of multi‐grade particles caused frequent clogging, impeding the passage of target single‐leaf particles. In contrast, the novel (right) drum exhibited reduced accumulation at the inlet small‐aperture section. The short spacing between “medium‐small” aperture sections allowed some single‐leaf particles to pass through adjacent medium apertures in advance, promoting axial particle diffusion, alleviating local accumulation, and facilitating smoother subsequent axial movement.

At *t* = 4.7 s, particles in the conventional drum had advanced to the large‐aperture section. Persistent entanglement and entrainment led to simultaneous sieving of single‐leaf and one‐bud‐one‐leaf particles, resulting in blurred classification boundaries and severe material mixing. The novel drum, however, showed significant improvements, with a large number of blue single‐leaf particles discharging in batches. Pre‐sieving weakened inter‐particle entanglement, enabling stable sieving of single‐leaf particles in the second small‐aperture section and orderly discharge of one‐bud‐one‐leaf particles from the second medium‐aperture section, resulting in clear classification and enhanced efficiency. These results validate the advantages of the novel aperture layout in improving screening efficiency and accuracy.

#### 3.1.2 Drum Inclination Angle of 5.5°

**FIGURE 7:**
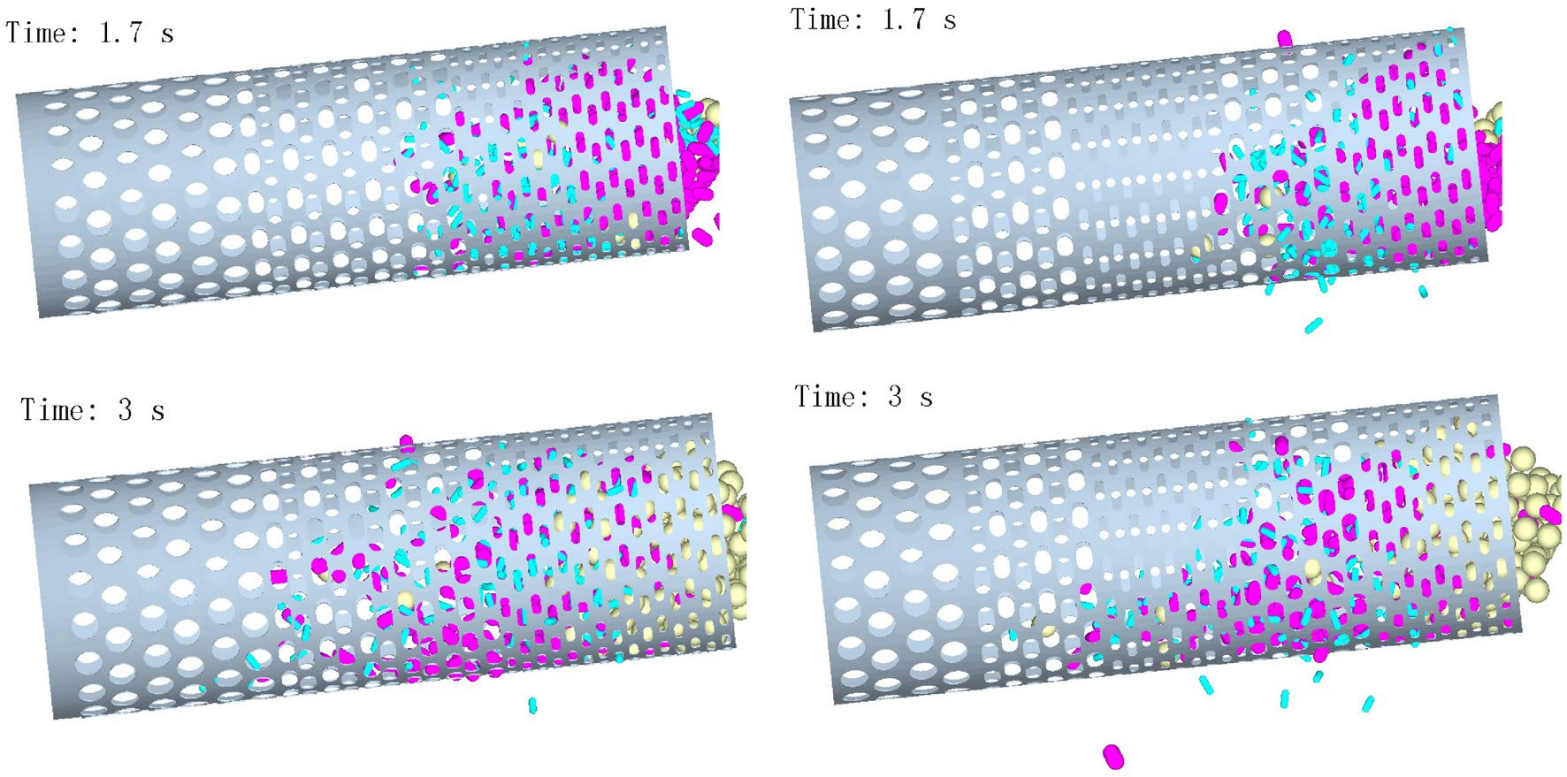
Particle motion when the inclination angle of the drum is 5.5°.

As shown in Figure 7, at *t* = 1.7 s, the increased inclination angle accelerated axial particle movement in the conventional drum, reducing accumulation but not eliminating entanglement and clogging. The novel drum exhibited further reduced inlet accumulation, with direct sieving of some single‐leaf particles. The combined effects of “medium‐small” aperture shunting and enhanced axial driving force resulted in faster particle diffusion and improved movement smoothness, with stable screening observed earlier than at 3°.

At *t* = 3 s, particles in the conventional drum had advanced to the large‐aperture section. Entanglement‐induced mixing remained prominent, and accelerated axial movement exacerbated blurred classification boundaries, with no improvement in accuracy. The novel drum demonstrated optimized performance, with a large number of blue single‐leaf particles discharging in batches at an earlier time than at 3°. Pre‐shunting and diffusion effectively weakened entanglement, enabling stable sieving of single‐leaf particles in the second small‐aperture section and orderly axial movement of one‐bud‐one‐leaf particles to the second medium‐aperture section for discharge. These results confirm the synergistic benefits of the novel aperture layout at 5.5°.

#### 3.1.3 Drum Inclination Angle of 8°

**FIGURE 8:**
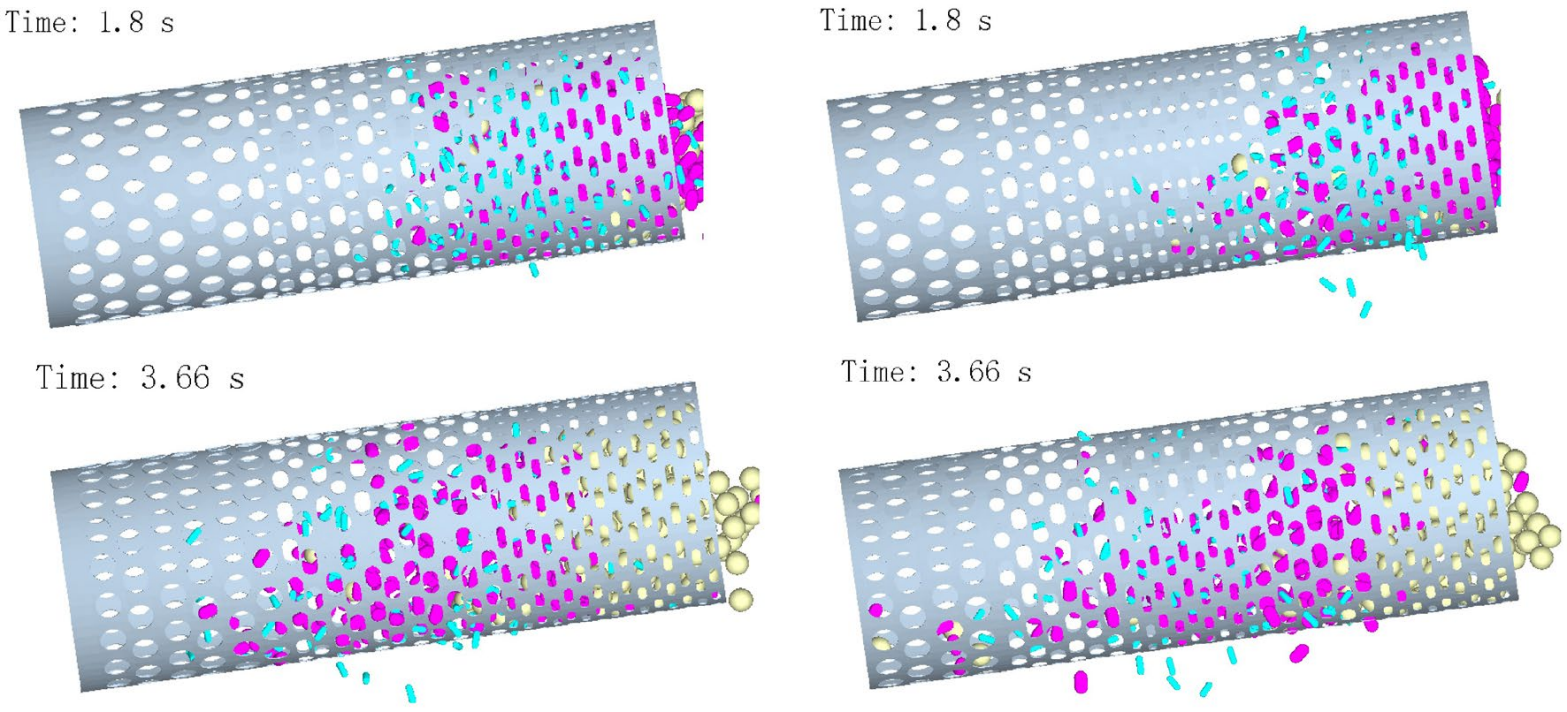
Particle motion when the inclination angle of the drum is 8°.

As shown in Figure 8, drum inclination angle of 8°, in the conventional drum, the steeper inclination angle further accelerated axial movement, reducing accumulation but not eliminating entanglement and clogging. The novel drum exhibited even lower inlet accumulation and earlier sieving of single‐leaf particles. Combined with the shunting effect of “medium‐small” apertures, axial particle diffusion was significantly enhanced, with smoother movement observed than at 5.5°.

At later simulation times, particles in the conventional drum advanced rapidly to the large‐aperture section, with entanglement‐induced mixing and blurred boundaries more severe than at 5.5°. The novel drum continued to show improved performance, with higher efficiency and earlier sieving times. These results validate the synergistic advantages of the novel layout at 8°.

#### 3.1.4 Drum Inclination Angle of 10°

**FIGURE 9:**
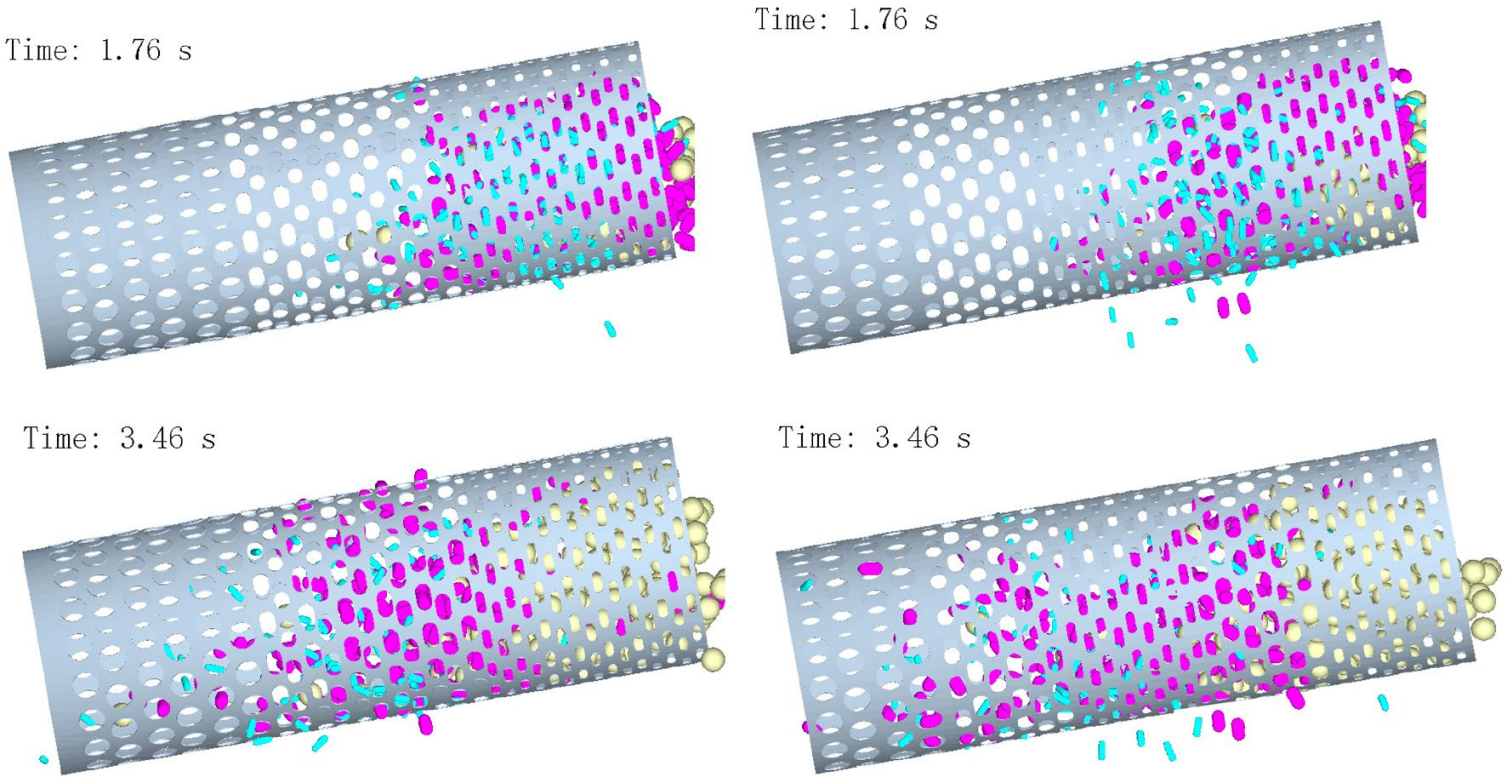
Particle motion when the inclination angle of the drum is 10°.

As shown in Figure 9, in the conventional drum, the steep 10° angle resulted in rapid axial movement, reducing accumulation but not eliminating entanglement and clogging. The novel drum exhibited minimal inlet accumulation and earlier sieving of single‐leaf particles, with faster axial diffusion and smoother movement than at 8°.

At later times, the conventional drum's entanglement‐induced mixing and blurred boundaries were further exacerbated by accelerated movement. The novel drum maintained superior performance, with higher efficiency and earlier sieving times, confirming the benefits of the novel aperture layout at 10°.

#### 3.1.5 Main Structure of the Rotary Drum Screen

Based on the above analysis, the “small‐medium‐small‐medium‐large” aperture layout effectively addresses particle entanglement and clogging through close‐range “medium‐small” shunting. When combined with inclination angles of 3°–10°, it optimizes axial particle movement smoothness and enables accurate single‐leaf and one‐bud‐one‐leaf classification. Its efficiency and accuracy significantly outperform the conventional layout, with performance advantages becoming more pronounced at higher inclination angles.

**FIGURE 10:**
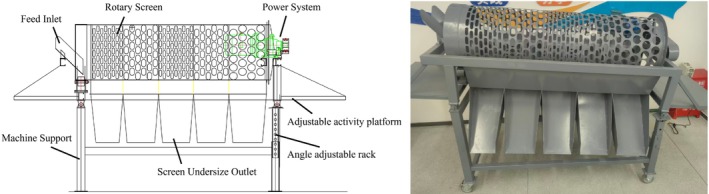
Main structural components of drum screen.

The angle‐adjustable rotary drum screen for fresh tea leaf classification is designed for grading multi‐grade leaves while removing small impurities such as sediment and broken branches. Its main structure, illustrated in Figure 10, comprises a feeding system, drum screen body, angle adjustment mechanism, screen cleaning device, transmission system, frame and support components, discharging system, and adjustable movable platform. The drum screen body is the core classification component, with precision‐punched apertures developed based on experimental tea leaf models. Aperture specifications can be customized for different tea varieties, and the novel “small‐medium‐small‐medium‐large” layout is employed in this design.

### 3.2 Experiments of Influencing Factors on Fresh Tea Leaf Drum Classification

#### 3.2.1 Single‐Factor Experiments

Tea separation efficiency is defined as the proportion of tea leaves sorted according to quality standards (e.g., shape and size), reflecting the accuracy and effectiveness of classification equipment (Zhang et al. 2025). The formula is:

(2) $$\varphi =\frac{A}{W}\times 100\%$$

where *A* is the number (or mass) of tea leaves meeting target standards post‐sorting; *W* is the total number (or mass) of sorted leaves.

#### 3.2.2 Single‐Factor Experiment of Drum Rotational Speed

**FIGURE 11:**
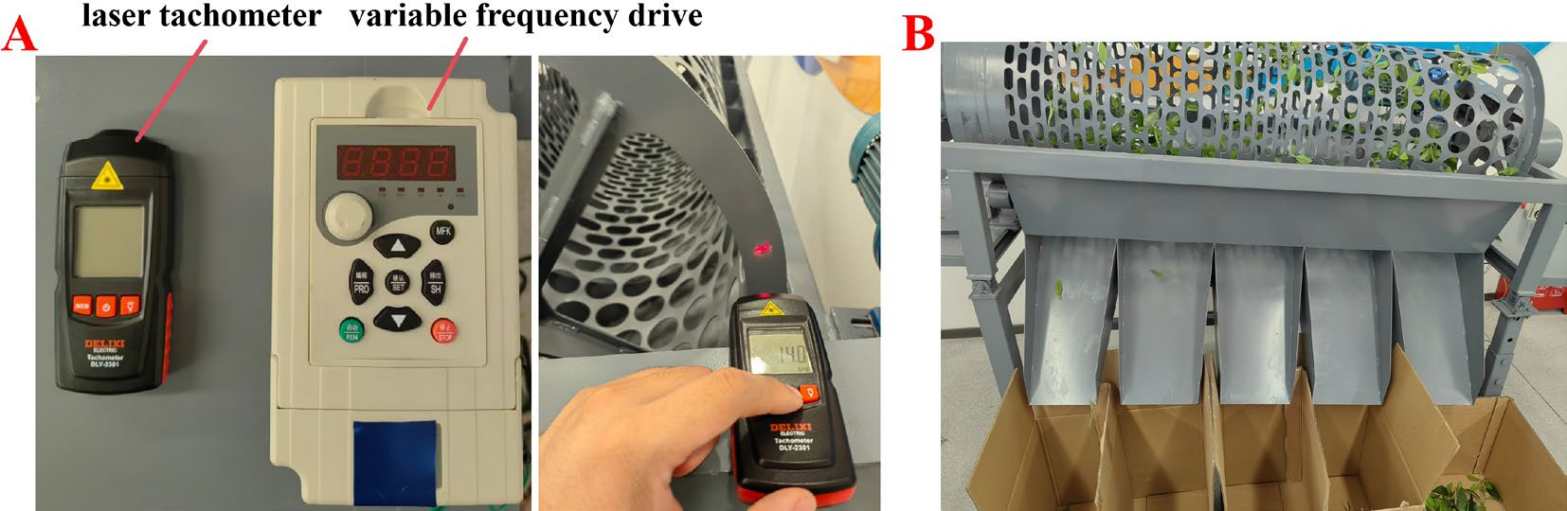
Roller speed measurement and screening test.

A single‐factor experiment was conducted to investigate the effect of drum rotational speed on sorting performance. The drum is driven by a motor, with speed adjusted via a frequency converter and calibrated using a laser tachometer (Figure 11). Rotational speeds of 7, 14, 21, and 28 r/min were tested to determine feasible operating ranges. The feed rate was set to a medium level (350 multi‐grade fresh tea leaves: 110 single‐leaf, 110 one‐bud‐one‐leaf, 130 one‐bud‐two‐leaf/multi‐leaf), and the inclination angle was fixed at 5.5°.

Separation efficiency was measured as the response variable, with three replicates per condition. Results (Table 2) show that average screening efficiency decreased continuously from 66.47% to 36.97% as rotational speed increased from 7 to 28 r/min, indicating that higher speeds reduce effective leaf‐screen contact, impairing sorting performance. In the low‐speed range (7–14 r/min), efficiency remained above 61% with low variability, indicating stable operation and effective sorting. At medium‐to‐high speeds (21–28 r/min), efficiency declined significantly, falling below 55% at 21 r/min and below 40% at 28 r/min. Thus, 7–14 r/min is identified as the optimal range, balancing high efficiency with acceptable throughput (lower at 7 r/min).

**TABLE 2:** Single‐factor experiment of drum rotational speed.

Drum rotational speed (r/min)	Test the screening efficiency			Average separation efficiency
	The first time	The second time	The third time	
7	71.5%	67.6%	60.3%	66.47% ± 5.69%
14	58.7%	63.7%	61.3%	61.23% ± 2.50%
21	49.7%	53.8%	50.2%	51.23% ± 2.24%
28	36.7%	43.3%	30.9%	36.97% ± 6.20%

#### 3.2.3 Single‐Factor Experiment of Drum Inclination Angle

A single‐factor experiment investigated the effect of drum inclination angle on sorting performance. The rotational speed was fixed at 14 r/min, and the feed rate at the medium level (350 leaves). Inclination angles of 0°, 3°, 5.5°, 8°, and 10° were tested.

Separation efficiency was measured as the response variable, with three replicates per condition. Results (Table 3) show that average screening efficiency increased with inclination angle from 0° to 5.5°, peaking at 5.5°, then gradually declined at 8° and 10°, indicating a non‐linear relationship. The optimal inclination angle was found to be approximately 5.5°, with 3°–8° identified as the feasible range for high, stable efficiency. At angles < 3°, insufficient gravitational driving force caused material accumulation at the drum bottom, with upper leaves blocked from effective screen contact. At angles > 8°, rapid axial movement reduced residence time, preventing full screening before discharge and causing leaf bouncing/splashing, resulting in reduced efficiency.

**TABLE 3:** Single‐factor experiment of drum inclination angle.

Drum tilt angle (°)	Test the screening efficiency			Average separation efficiency
	The first time	The second time	The third time	
0	28.4%	37.6%	30.3%	32.10% ± 4.86%
3	52.6%	59.7%	48.3%	53.53% ± 5.76%
5.5	58.7%	63.7%	61.3%	61.23% ± 2.50%
8	54.3%	58.5%	63.1%	58.63% ± 4.40%
10	30.6%	27.4%	32.9%	30.30% ± 2.76%

#### 3.2.4 Single‐Factor Experiment of Drum Feed Rate

**FIGURE 12:**
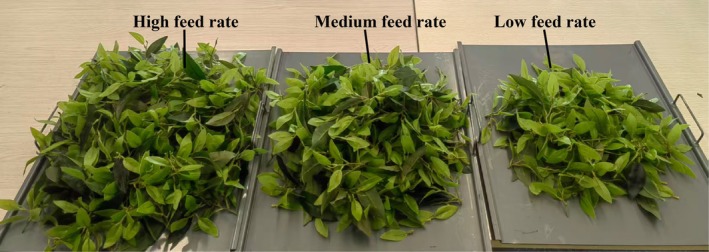
Tea leaves feed rate.

A single‐factor experiment investigated the effect of feed rate on sorting performance. The rotational speed was fixed at 14 r/min, and the inclination angle at 5.5°. Feed rates of low (150 leaves), medium (350 leaves), and high (550 leaves) were tested (Figure 12).

Separation efficiency was measured as the response variable, with three replicates per condition. Results (Table 4) show that average screening efficiency decreased continuously as feed rate increased from low to high. At low feed rates, thin, evenly distributed material layers ensured sufficient leaf‐screen contact, yielding the highest efficiency with low variability, though throughput was limited. At medium feed rates, efficiency was only slightly lower, indicating the equipment maintains high performance under normal operating loads. At high feed rates, efficiency declined by approximately 15% compared to medium rates due to material accumulation, inter‐leaf shielding, reduced effective screen contact, and accelerated axial movement with insufficient residence time.

**TABLE 4:** Single‐factor experiment of drum feed rate.

Feed rate	Test the screening efficiency			Average separation efficiency
	The first time	The second time	The third time	
Low	63.5%	66.7%	58.3%	62.83% ± 4.24%
Medium	58.7%	63.7%	61.3%	61.23% ± 2.50%
High	46.2%	41.3%	50.9%	46.13% ± 4.80%

### 3.3 Response Surface Methodology (RSM) Experimental Analysis

#### 3.3.1 Response Surface Factors

Based on single‐factor experiment results, three levels of each factor with optimal performance were selected for RSM experiments. Using Box–Behnken central composite design principles, three significant factors were included (Zhang and Wang 2025), as detailed in Table 5.

**TABLE 5:** Response surface experimental design.

Factor	Level		
	−1	0	1
A: Drum rotational speed (r/min)	7	14	21
B: Drum tilt angle (°)	3	5.5	8
C: Feed rate (leaf)	150	350	550

#### 3.3.2 Analysis of Response Surface Experimental Results of Drum Separation Efficiency

A three‐factor, three‐level RSM design was employed, with five central replicate experiments (Zhu 2025; Gao et al. 2024; Wu et al. 2026). The experimental scheme and results for drum separation efficiency are presented in Table 6.

**TABLE 6:** Experimental scheme and results.

No.	A: Drum rotational speed (r/min)	B: Drum tilt angle (°)	C: Feed rate (leaf)	Y: Separation efficiency (%)
1	7	3	350	54.4
2	21	3	350	46.6
3	7	8	350	44.5
4	21	8	350	41.1
5	7	6	150	43.5
6	21	6	150	40.6
7	7	6	550	53.3
8	21	6	550	43.5
9	14	3	150	42.5
10	14	8	150	31.7
11	14	3	550	48.3
12	14	8	550	43.7
13	14	6	350	63.3
14	14	6	350	62.3
15	14	6	350	62.5
16	14	6	350	63.2
17	14	6	350	62.1

Analysis of variance (ANOVA) conducted via Design‐Expert 13.0 software (Table 7) revealed a model *p*‐value < 0.0001 (highly significant), indicating the regression model effectively describes variations in separation efficiency. The *F*‐value of 257.75 confirms strong explanatory power and reliable fitting (Guo et al. 2026; Feng et al. 2023; Liu et al. 2022). Main effects of drum rotational speed (A), inclination angle (B), and feed rate (C) were all highly significant (*p* < 0.0001), with feed rate and inclination angle exhibiting slightly stronger effects than rotational speed based on *F*‐values and mean squares.

**TABLE 7:** Analysis of variance of the regression model.

Source	Sum of squares	Degree of freedom	Mean square	*F*‐value	*p*
Model	1557.73	9	173.08	257.75	< 0.0001
A‐Drum rotational speed	71.40	1	71.40	106.33	< 0.0001***
B‐Drum tilt angle	118.58	1	118.58	176.59	< 0.0001***
C‐Feed rate	116.28	1	116.28	173.17	< 0.0001***
AB	4.84	1	4.84	7.21	0.0313**
AC	11.9	1	11.9	17.73	0.0040***
BC	9.61	1	9.61	14.31	0.0069***
A^2^	160.68	1	160.68	239.29	< 0.0001***
B^2^	408.72	1	408.72	608.67	< 0.0001***
C^2^	535.50	1	535.50	797.478	< 0.0001***
Residual	4.70	7	0.6715		
Lack of Fit	3.53	3	1.18	4.03	0.1057*
Pure Error	1.17	4	0.2920		
Cor Total	1562.44	16			

*Note:* ***Extremely significant differences (*p* < 0.01); **Significant differences (0.01 < *p* < 0.05); *Non‐significant differences (*p* > 0.05).

The Lack of Fit test yielded a *p*‐value of 0.1057 (*p* > 0.05), indicating no significant lack of fit (Chen et al. 2019). This confirms the quadratic model accurately reflects the inherent relationships between factors and separation efficiency, rather than fitting experimental noise, validating the model's goodness of fit.

Interaction effects: AB was significant (*p* < 0.05), while AC and BC were highly significant (*p* < 0.01), indicating the need to consider both individual factors and their synergistic effects during optimization. Quadratic terms A2, B2, and C2 were all highly significant (*p* < 0.0001), confirming non‐linear relationships between factors and separation efficiency, supporting the use of a quadratic model.

Through the above analysis, the multiple regression model was established as:

(3) $$\begin{array}{c} Y=62.68-2.99A-3.85B+3.81C+1.1\mathrm{AB}-1.72\mathrm{AC}\\ \;+1.55\mathrm{BC}-6.18A^{2}-9.85B^{2}-11.28C^{2} \end{array}$$

**FIGURE 13:**
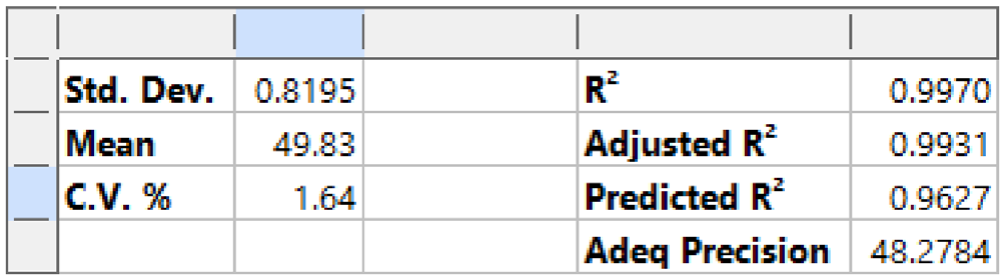
Regression model fitting statistics.

Model fitting statistics (Figure 13) show a coefficient of determination *R* ^2^ = 0.9970, indicating the model explains over 99.7% of response value variations, with high correlation between predicted and actual values. The Predicted *R* ^2^ of 0.9627 reflects strong predictive ability, while the Adjusted *R* ^2^ of 0.9931 confirms the model effectively distinguishes factor effects on separation efficiency, with a high signal‐to‐noise ratio. The difference between Predicted and Adjusted *R* ^2^ is 0.0304 (< 0.2), indicating reliable prediction of new experimental results. The coefficient of variation (CV) is 1.64%, well below the 10% threshold, indicating stable experimental operation and minimal data variability (Zhang et al. 2026; Li et al. 2026; Yang et al. 2026).

**FIGURE 14:**
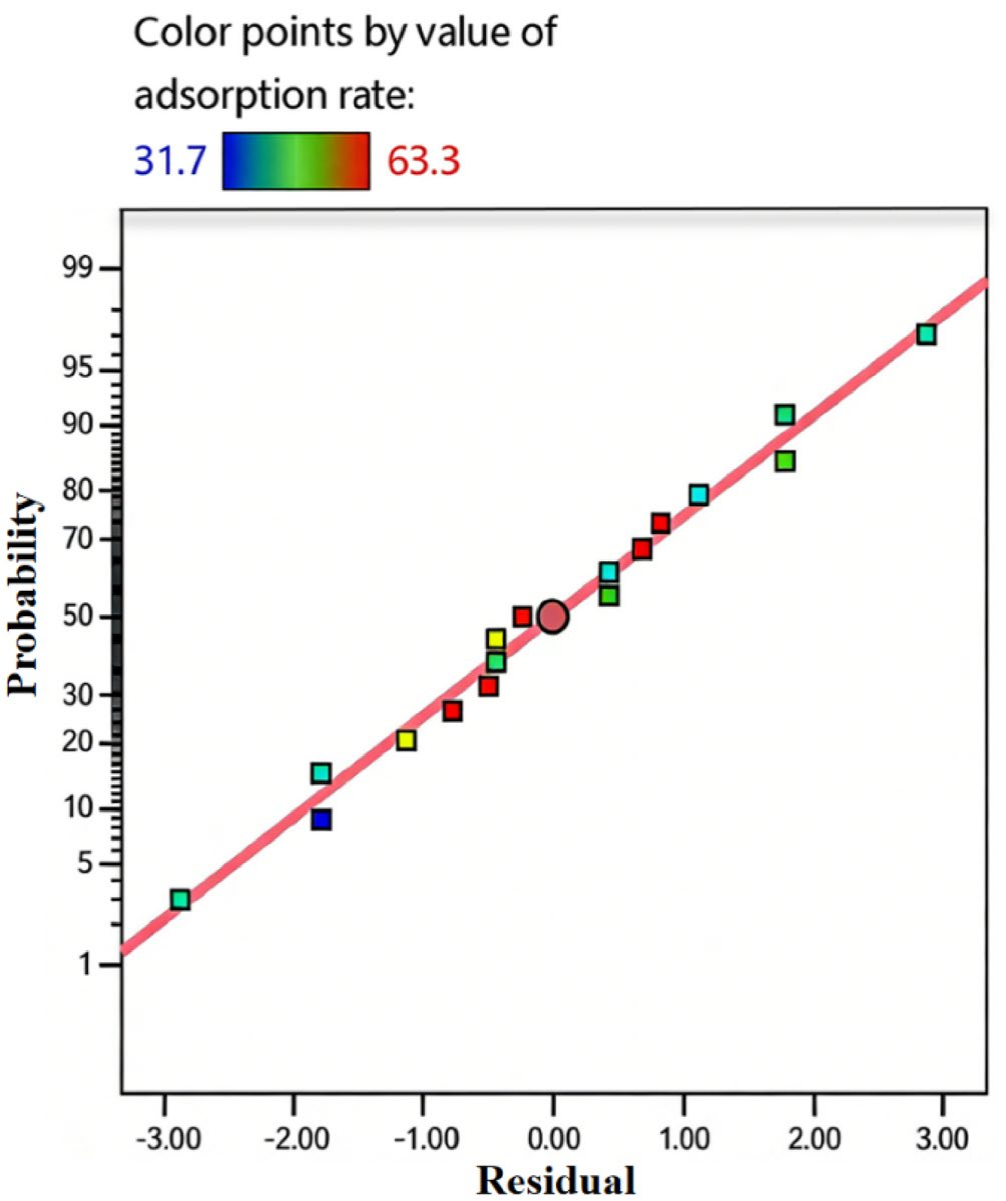
Normal probability plot of residuals.

**FIGURE 15:**
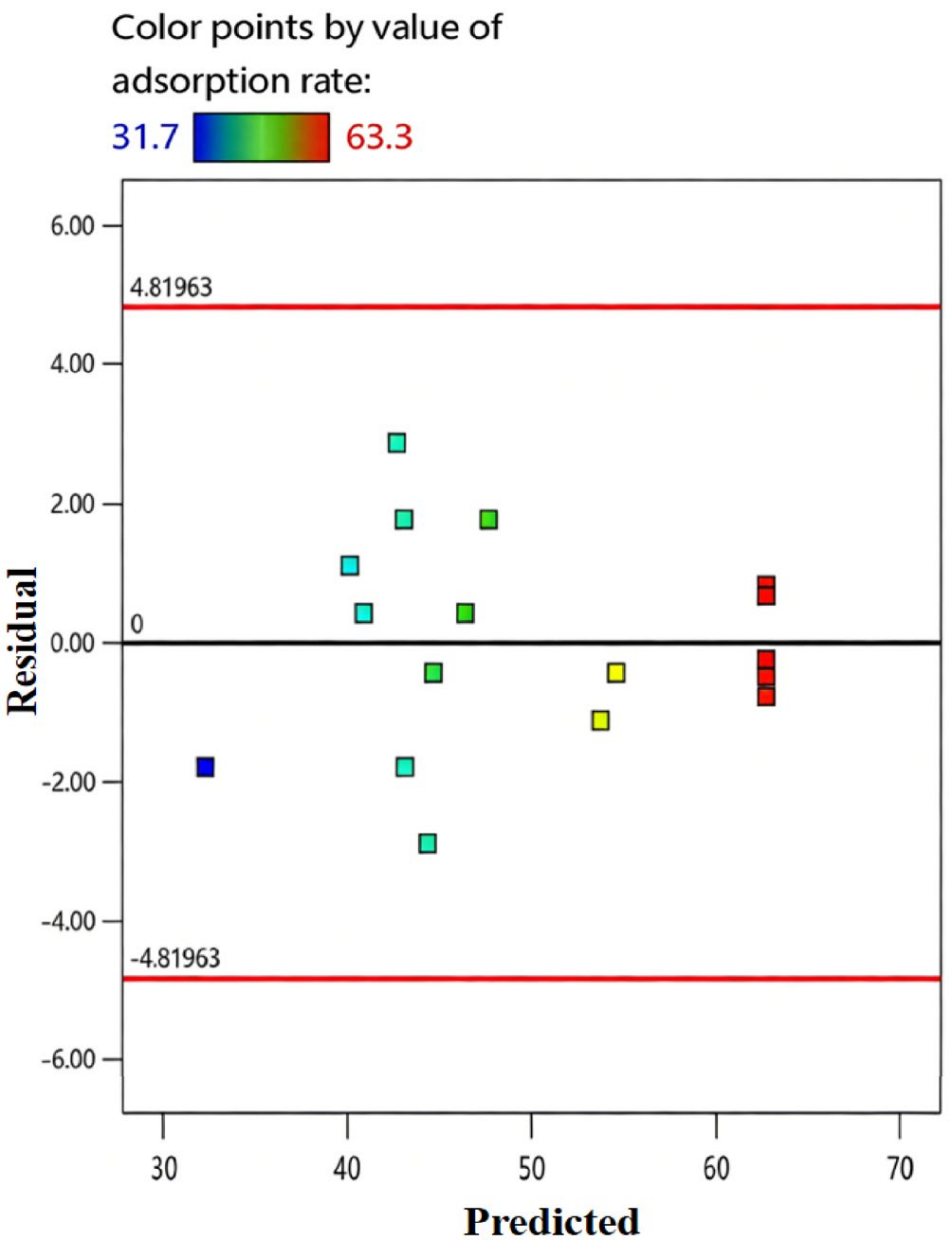
Residual‐predicted value scatter plot.

To further validate model applicability, normal probability plots of residuals and residual‐predicted value scatter plots were generated (Figures 14 and 15). Residuals followed a normal distribution, with no significant deviations, indicating no systematic errors. Residuals were randomly distributed around the zero baseline without funnel‐shaped or linear trends, confirming model homoscedasticity. These results demonstrate the quadratic model accurately describes the nonlinear relationships between factors and separation efficiency, validating its applicability.

#### 3.3.3 Analysis of the Influence of Interactive Factors on Experimental Indicators

##### 3.3.3.1 Interaction of Factors A and B

**FIGURE 16:**
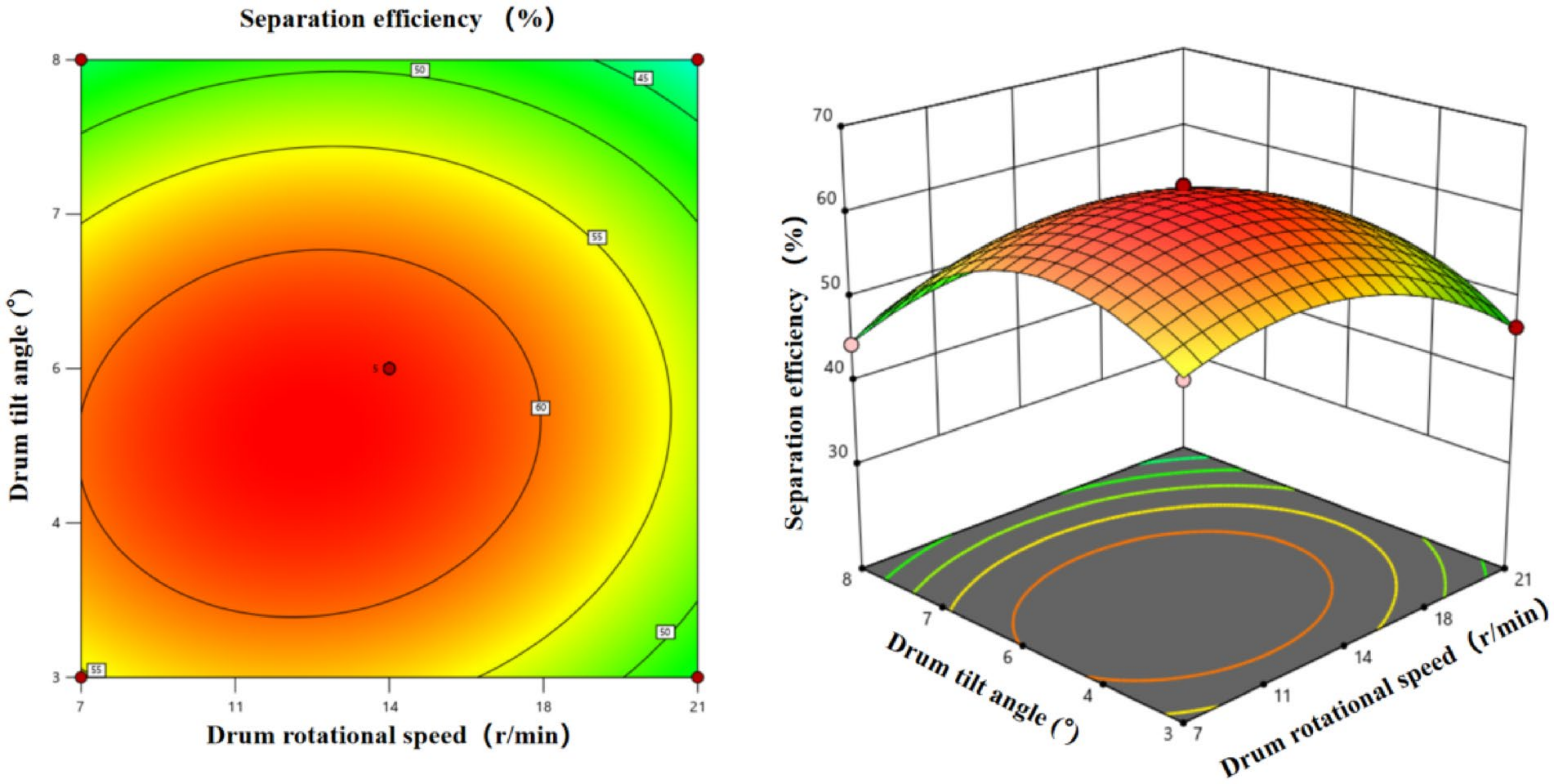
Contour plot and response surface plot of the interaction of factors A and B on the drum separation efficiency of multi‐scale fresh tea leaves.

Figure 16 illustrates the effects of the interaction between rotational speed (A) and inclination angle (B) on separation efficiency. Contour plots show concentric circles with the highest efficiency (60%–63%) centered at 14 r/min and 5.5°. Deviations from these levels result in reduced efficiency, reflecting a quadratic nonlinear relationship. The 3D response surface plot shows a “hill” shape with the peak at the center, exceeding 60% efficiency, which decreases uniformly to 40%–50% at the periphery, consistent with contour plot trends. This confirms the highly significant quadratic effects of A^2^ and B^2^, demonstrating clear optimal levels for both factors.

##### 3.3.3.2 Interaction of Factors A and C

**FIGURE 17:**
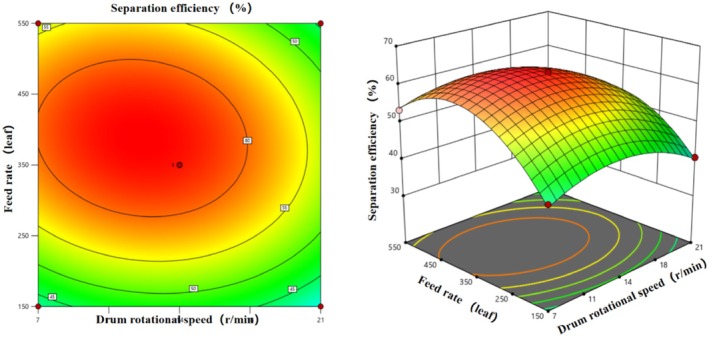
Contour plot and response surface plot of the interaction of factors A and C on the drum separation efficiency of multi‐scale fresh tea leaves.

Figure 17 illustrates the effects of the interaction between rotational speed (A) and feed rate (C) on separation efficiency. Contour plots show concentric circles with the highest efficiency centered at medium levels of both factors. The response surface plot is a typical quadratic “hill” shape, peaking at 14 r/min and medium feed rate (> 60% efficiency), decreasing to 40%–50% at the periphery. At a fixed medium feed rate, efficiency follows a quadratic trend (increasing then decreasing) with increasing rotational speed (7–21 r/min), reflecting the significant A^2^ effect. At a fixed 14 r/min, efficiency similarly follows a quadratic trend with increasing feed rate (low to high), reflecting the significant C^2^ effect.

##### 3.3.3.3 Interaction of Factors B and C

**FIGURE 18:**
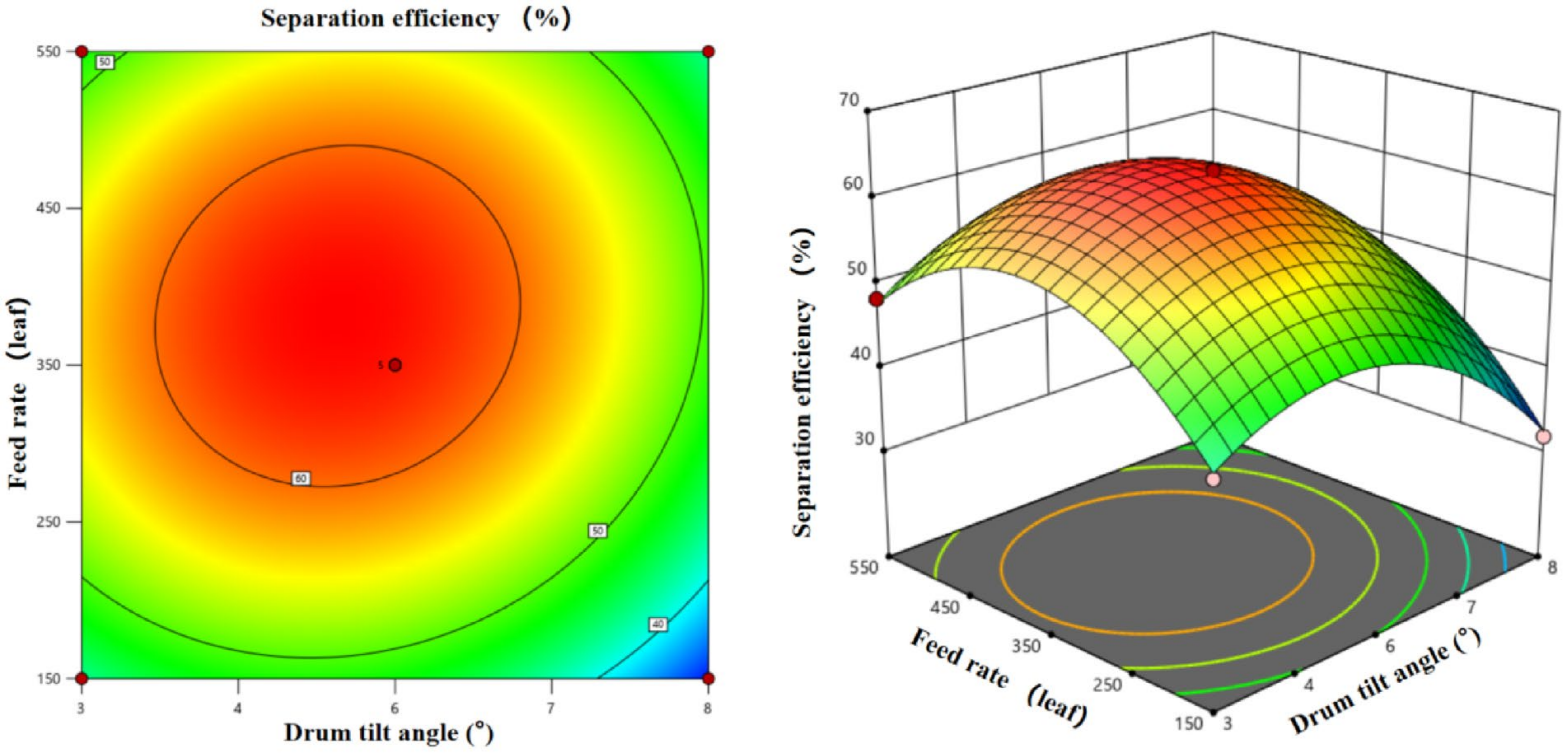
Contour plot and response surface plot of the interaction of factors B and C on the drum separation efficiency of multi‐scale fresh tea leaves.

Figure 18 illustrates the effects of the interaction between inclination angle (B) and feed rate (C) on separation efficiency. Contour plots show concentric circles with the highest efficiency centered at 5.5° and medium feed rate. The response surface plot is a quadratic “hill” shape, peaking at > 60% efficiency, decreasing to 40%–50% at the periphery. At a fixed medium feed rate, efficiency follows a quadratic trend with increasing inclination angle (3°–8°), reflecting the significant B^2^ effect. At a fixed 5.5°, efficiency follows a quadratic trend with increasing feed rate (low to high), reflecting the significant C^2^ effect. The uniform slope of the surface indicates similar influences of inclination angle and feed rate, with synergistic regulation requiring both factors to be at optimal levels for maximum efficiency.

### 3.4 Mechanism Analysis of the Interaction Effects of Various Factors

The core process of rotary drum screening for fresh tea leaves involves the dynamic coupling of three key stages: axial transport, radial tumbling, and stratification, and screen penetration. The three factors—rotational speed, inclination angle, and feed rate—do not act independently but exert synergistic or antagonistic effects by regulating these stages, with statistical laws reflecting the quantitative manifestation of physical screening mechanisms.

#### 3.4.1 Interaction Between Drum Rotational Speed and Drum Inclination Angle (*P* = 0.0313)

From The drum inclination angle determines axial velocity and effective screening duration via gravitational components, while rotational speed controls radial tumbling frequency, throwing height, and stratification via centrifugal force. At low inclination angles (3°), weak axial driving force results in slow movement and long residence times, requiring moderate rotational speeds to break accumulation and promote stratification. Excessively low speeds fail to lift materials, leading to compacted layers and blocked penetration; excessively high speeds cause materials to adhere to the drum wall, eliminating throwing opportunities. At high inclination angles (8°), rapid axial movement compresses screening duration, amplifying the influence of rotational speed: high speeds accelerate material passage with no effective penetration time, while low speeds fail to stratify materials. Thus, balanced moderate levels of both factors are required to maximize efficiency.

#### 3.4.2 Interaction Between Drum Rotational Speed and Feed Rate (*P* = 0.0040)

The feed rate determines material layer thickness, inter‐particle entanglement probability, and screen contact rate, directly influencing clogging. At high feed rates, thick layers and strong entanglement impose strict requirements on rotational speed: low speeds fail to lift and throw materials, leaving only the bottom layer in contact with the screen; high speeds cause layers to adhere to the drum wall, intensifying entanglement and clogging. Moderate speeds break entanglement, enable stratification, and allow ordered penetration. At low feed rates, thin layers and weak entanglement reduce the impact of rotational speed, resulting in significant interaction effects.

#### 3.4.3 Interaction Between Drum Inclination Angle and Feed Rate (*p* = 0.0069)

The feed rate and inclination angle jointly regulate axial material distribution uniformity and clogging risk. At high feed rates, materials are prone to accumulation at the inlet, amplifying the inclination angle's effect: angles < 3° result in insufficient axial driving force, causing thick layers and severe entanglement/clogging; angles > 8° accelerate movement, reducing residence time and entraining fine particles before penetration. Moderate angles (e.g., 5.5°) balance driving force and residence time, preventing accumulation and ensuring effective screening. At low feed rates, minimal accumulation reduces the inclination angle's impact, resulting in significant interaction effects.

### 3.5 Parameter Optimization and Experimental Verification

**FIGURE 19:**
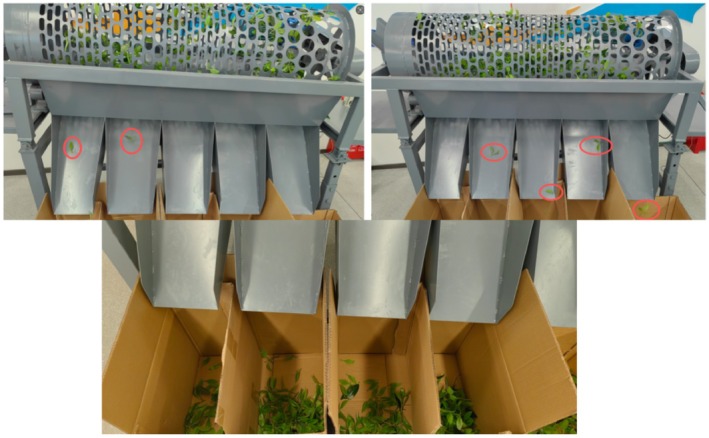
Optimization experiment.

Using Design‐Expert's Optimization function, parameters were optimized to maximize separation efficiency. The optimal conditions were identified as: rotational speed 13 r/min, inclination angle 5.5° (process constraint), and feed rate 300 multi‐grade fresh tea leaves. Three replicate experiments yielded an average separation efficiency of 70.49%, as shown in Figure 19.

While results exceed previous tests, efficiency remains below ideal due to tea leaves adhering to the screen surface. Future work will incorporate an airflow field to reduce adhesion. Additionally, single‐batch tests using actual fresh tea leaves yielded an average breakage rate of 2.17% and a bud‐and‐leaf fracture rate of 1.32%, both well below the 5% threshold for industry‐standard equipment. This confirms the design's ability to preserve tea quality while achieving high‐efficiency sorting due to the smooth inner drum surface and absence of internal guide plates.

Compared to commercial rotary drum screens widely used in primary tea processing, which typically achieve 45%–55% efficiency in continuous multi‐grade leaf production and suffer from frequent clogging and cleaning shutdowns (Xue et al. 2020; Li et al. 2024), the optimized equipment achieved 70.49% efficiency—an absolute increase of 20.49 percentage points (relative increase of 40.98%) over the industry average of 50%. Clogging issues were significantly mitigated, reducing cleaning shutdown frequency and improving continuous production performance.

### 3.6 Research Limitations

The DEM simulation adopted simplified ellipsoidal particles to replace real flexible fresh tea leaves for higher computational efficiency. Despite a relative error of only 4.03% within the permissible range, the model fails to fully reflect the flexible deformation, stem‐leaf interlocking, and severe entanglement of actual tea leaves. This limitation only affects the absolute prediction accuracy and does not change the core advantage of the innovative alternating sieve layout in reducing clogging and improving grading accuracy.

The experimental fresh tea samples are single‐variety, from one tea plantation and harvested in a fixed period. Since tea physical properties differ greatly with variety, harvest season, and moisture content, the optimized parameters have limited universal applicability. Nevertheless, the proposed DEM‐RSM coupling method and the interaction rule of influencing factors can be widely applied to the structural design and parameter optimization of tea leaf grading equipment.

The existing rotary drum screen has the defect of fresh tea leaves adhering to the sieve surface, which restricts further improvement of separation efficiency. This issue only influences the optimal efficiency value, without undermining the reliability of parameter optimization results and the superiority verification of the new sieve hole arrangement.

## 4 Conclusion

1. The innovative alternating aperture layout of small‐medium‐small‐medium‐large effectively alleviates particle entanglement and sieve clogging of fresh tea leaves via aperture flow distribution. Within the drum inclination range of 3°–10°, it presents remarkably higher screening efficiency and grading accuracy than the conventional sequential layout, with its superiority becoming more prominent at a larger inclination angle.
2. A quadratic regression model for separation efficiency was established with drum rotational speed, inclination angle, and feed rate as influencing factors. The model is highly significant with high fitting accuracy, which can precisely reflect the nonlinear effects of single factors and their interactions on separation efficiency. The inclination angle and feed rate exert slightly greater influences than rotational speed.
3. The optimal operating parameters are determined as the rotational speed of 13 r/min, inclination angle of 5.5°, and feed rate of 300 tea leaves, achieving an average separation efficiency of 70.49%. The optimized parameters outperform traditional empirical settings with excellent experimental stability, which can provide theoretical and technical support for the standardization of primary tea processing.

## Author Contributions

**Xu Zhang:** writing – original draft, writing – review and editing, validation, resources, visualization. **Zhongyou Zhou:** software, resources, visualization. **Rongyang Wang:** software, resources, visualization.

## Funding

This work was supported by the Huzhou Public Welfare Application Research Project (2025GY049), General scientific research project of Zhejiang Education Department (Y202558782), and “Mirror Lake Talents” Project of Huzhou Vocational and Technical College (JH2023007).

## Conflicts of Interest

The authors declare no conflicts of interest.

## Data Availability

The data that support the findings of this study are available from the corresponding author upon reasonable request.
